# Rural–Urban Disparities in Perinatal Smoking in the United States: Trends and Determinants

**DOI:** 10.3390/ijerph22060895

**Published:** 2025-06-04

**Authors:** Patricia Da Rosa, Matthias Richter

**Affiliations:** 1 Social Determinants of Health, TUM School of Medicine and Health, Department of Health and Sport Sciences, Technical University of Munich, 80809 Munich, Germany; richter.matthias@tum.de; 2 Preventive Pediatrics, TUM School of Medicine and Health, Department of Health and Sport Sciences, Technical University of Munich, 80809 Munich, Germany

**Keywords:** pregnancy, PRAMS, smoking, inequalities, tobacco use, rural health

## Abstract

Objective: To examine trends in perinatal smoking across rural and urban areas and investigate whether structural and intermediary health factors explain rural-urban disparities. Methods: This cross-sectional study used data from the Pregnancy Risk Assessment Monitoring System (PRAMS) collected between 2009 and 2021 in the United States. Perinatal smoking patterns were based on self-reported smoking before, during, and after pregnancy. Weighted prevalence estimates with 95% confidence intervals (CIs) were calculated for persistent smoking and cessation, stratified by rural–urban residence. Temporal trends were analyzed using logistic regression. Multivariable weighted logistic regression was performed on Phase 8 data (2016–2021) to examine associations between rural–urban status and perinatal smoking patterns, adjusting for maternal age, year of delivery, region, and structural (e.g., education, Tobacco 21 policy) and intermediary (e.g., perinatal stressors) health determinants. All analyses accounted for the complex survey design. Results: Although perinatal smoking declined over time, prevalence remained consistently higher among rural mothers. From 2009 to 2021, persistent smoking decreased significantly in both rural and urban areas (*p* < 0.001). Smoking cessation rates remained stable (*p* = 0.087), with no significant difference by rural–urban status (*p* = 0.475). After adjustment, rural women were 45% more likely to smoke persistently than urban women (OR = 1.45, 95% CI: 1.35–1.56) and 26% less likely to quit smoking. Conclusions: While perinatal smoking declined overall, rural mothers remained more likely to smoke throughout pregnancy. Structural and intermediary determinants partially explained this persistent rural–urban disparity.

## 1. Introduction

Perinatal smoking remains the leading preventable cause of adverse health outcomes for both mothers and infants. Its short and long-term consequences are severe, including stillbirth, miscarriage, low birth weight (LBW), sudden infant death syndrome (SIDS), neonatal death, and preterm birth [1,2,3,4]. Additionally, perinatal smoking has been associated with reduced breastfeeding duration, cognitive impairments, and behavioral and emotional disorders in the offspring [5,6,7].

Although perinatal smoking has declined globally, with the latest prevalence at 1.7%, it remains a public health problem due to considerable geographical differences both between and within countries. Europe has the highest prevalence at 8.1%, followed by the Americas at 5.9%, while regions such as the Eastern Mediterranean show substantially lower rates (0.9%) [8]. These disparities are also evident within regions, particularly between rural and urban areas, though evidence among pregnant women remains limited. For instance, in a study of six Western European countries, the overall smoking prevalence was found to be higher in urban than in rural areas [9]. Conversely, multiple studies from the United States (U.S.) consistently show higher smoking rates in rural populations [10,11,12]. Among pregnant women specifically, data from the U.S. National Survey on Drug Use and Health from 2002 to 2019 showed that 25.3% of rural women smoked during pregnancy compared to 12.0% in urban areas. Smoking cessation rates were also lower in rural pregnant women, with only 21.5% quitting, compared to 33.0% in urban areas during the same period [10].

While psychological frameworks such as the Theory of Planned Behavior [13] and Self-Determination Theory [14] help explain individual smoking behaviors, they do not fully account for significant geographic disparities in perinatal smoking, which are shaped by socioeconomic, cultural, and policy contexts [8]. To address these complexities, the World Health Organization’s Social Determinants of Health (SDOH) framework emphasizes the role of structural, contextual, and intermediary factors in shaping health inequities throughout the life course [15]. Applying this lens, a Brazilian longitudinal study found that although the prevalence of smoking during pregnancy declined over three decades, reductions were mainly observed among higher-income, white women, indicating persistent social inequalities [16]. In rural areas, structural disadvantages, such as higher poverty rates, lower insurance coverage, and limited access to primary healthcare, can also contribute significantly to these disparities [17]. Additionally, public health programs in these regions are often underfunded [18]. These challenges are further exacerbated by psychosocial problems, including depression and perinatal stress [19], exposure to secondhand smoke, cultural norms, and limited access to cessation services [12,17,20,21], all of which make quitting more challenging for women residing in these settings.

Despite growing awareness of these disparities, studies examining how structural and intermediary factors contribute to geographic differences in perinatal smoking remain limited, especially in the post-COVID-19 context [17,22]. Examining perinatal smoking through an SDOH approach is critical for developing targeted interventions, particularly for vulnerable populations like pregnant women in rural areas. Additionally, examining patterns of perinatal smoking (e.g., smoking persistence) is needed to guide the implementation of tobacco control measures among high-risk groups. Leveraging data from the Pregnancy Risk Assessment Monitoring System (PRAMS), this study aims to (i) update and expand the literature on the trends in perinatal smoking, including smoking cessation, by rural and urban status and (ii) investigate the association between perinatal smoking and rural–urban areas of residency, while accounting for structural and intermediary health determinants.

## 2. Methods

### 2.1. Study Design and Setting

This cross-sectional study used 2009–2021 data from the Pregnancy Risk Assessment Monitoring System (PRAMS), a well-established, population-based surveillance system developed to monitor specific maternal health behaviors and outcomes of U.S. women who delivered live infants. PRAMS follows a standardized data collection methodology designed by the Centers for Disease Control and Prevention (CDC). Data collection includes self-administered surveys sent to mothers 2 to 6 months postpartum, targeting their health and behavior outcomes and experiences before, during, and after pregnancy. For non-respondents to the mailed survey, a follow-up telephone interview is conducted. On average, PRAMS sites select between 1200 and 3600 mothers annually The inclusion criteria consist of resident women who recently gave birth to a live-born infant within their state during the surveillance year. Women are sampled between 2 and 6 months postpartum, and only one infant from multiple gestations is randomly selected for inclusion. Exclusion criteria included participants who were under the age of 14, state residents who gave birth out of state, non-residents who gave birth in the state, participants missing information (e.g., last name or mailing address), participants whose birth certificates were processed more than four months after the birth, and infants who were adopted [23].

The study received approval from the CDC PRAMS working group and the participating sites. As the data was de-identified, it did not meet the federal definition of human subject research (Code of Federal Regulations, 45 CFR 46.102) per the University Institutional Review Board.

### 2.2. Sample

**Figure 1:**
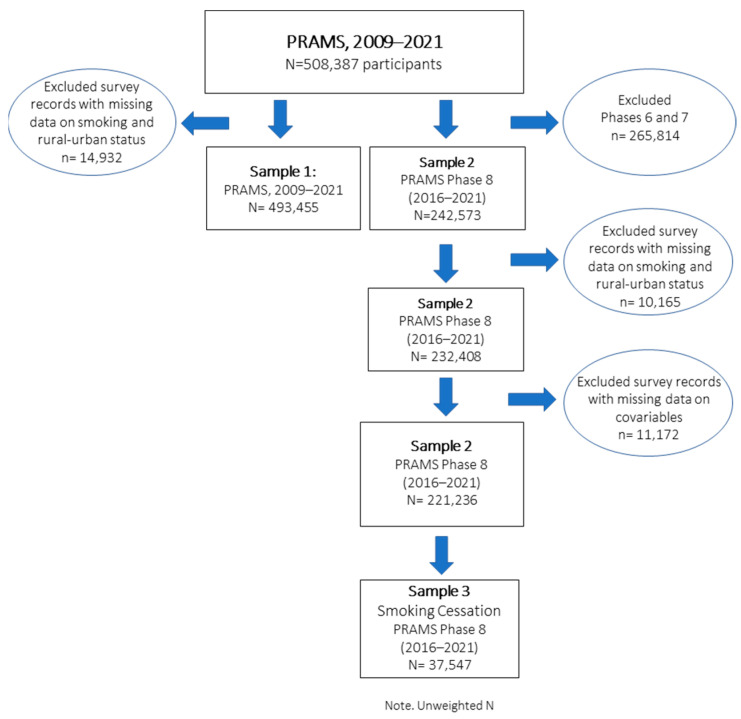
Flowchart of PRAMS participants and final study population.

To examine temporal trends in perinatal smoking, 493,455 women (unweighted) with valid data on rural–urban residency areas and perinatal smoking from 2009 to 2021 were included in the analysis, representing 97% of the overall sample and 47 PRAMS sites (Sample 1). Of the total participants, 103,633 (21.0%) resided in rural areas, while 389,822 (79.0%) lived in urban areas. To examine the association between perinatal smoking and rural–urban residency while accounting for health determinant factors and to methodological changes in the data collection (e.g., household income, federal benefit program participation) across survey phases, we restricted our analysis to a subsample (Sample 2) which included surveys from 2016 to 2021 (unweighted *N* = 232,408). To examine patterns in smoking cessation, Sample 3 included only participants who reported smoking before and or during pregnancy (Figure 1).

### 2.3. Measures

#### 2.3.1. Cigarette Smoking and Cessation

Perinatal smoking was based on self-reported questions from the PRAMS survey, which included smoking status at three key points: 3 months prior to pregnancy, during the last 3 months of pregnancy, and 2–6 months after delivery. To examine perinatal smoking patterns, we examined: non-smokers (no smoking at any time), smokers during pregnancy, and persistent smokers (smoked before, during, and after pregnancy). Finally, an additional variable was created to assess smoking cessation during pregnancy. It was defined as the proportion of women who reported not smoking any cigarettes during pregnancy, divided by the total number of women who smoked cigarettes three months before pregnancy (Table S1, Supplementary Material).

#### 2.3.2. Rural vs. Urban

Rural and urban classifications were based on the mother’s county of residence. Counties were initially grouped into six categories based on urbanization level and then further categorized into rural and urban areas using the National Center for Health Statistics (NCHS) categorization [24]. Thus, this study defined rural areas as micropolitan and noncore counties and urban areas as metropolitan statistical areas. This classification is frequently used in studies to examine the relationship between the urbanization level of residence and health outcomes [25].

To account for regional differences, states were categorized into the Midwest, Northeast, South, and West, following the US Census [26].

#### 2.3.3. Social Determinants of Health (SDOH) Factors

Following the WHO conceptual SDOH framework, we examined structural and intermediary health determinants. As a reflection of structural determinants, maternal education was self-reported and grouped into three levels: less than 12 years, 12 years, and more than 12 years of formal education. Similarly, household income was further categorized into 12 to 4 categories (≤$24,000, $24,001–$60,000, $60,001+, missing/not reported). Race/ethnicity was categorized as non-Hispanic White, non-Hispanic Black, Hispanic, or others (e.g., American Indian/Alaskan Native, Asian/Pacific Islander, Chinese, Japanese, Filipino, Hawaiian, and Other Asian), and missing/not reported. Intermediary determinants included age, marital status, participation in social programs, pregnancy experience (parity), and exposure to perinatal stressors. Marital status (ever married) was assessed as a proxy of social support and was categorized as married and non-married. During pregnancy, access to the supplemental nutritional program for Women, Infants, and Children (WIC) was also assessed. WIC provides vital support to low-income women, improving access to nutrition, healthcare, and smoking cessation resources. Parity, the number of times a woman has given birth to a child, thus a measurement of previous pregnancy experience, was categorized as 0, 1, 2, and 3 or more. Pregnancy intention was categorized into three groups: planned, mistimed (either later or sooner than desired), and unsure (which includes unplanned pregnancies). Self-reported depression during pregnancy was assessed by asking participants if they experienced depression during their most recent pregnancy, and responses were categorized as yes or no. Exposure to perinatal stressors was assessed by asking mothers whether a husband or partner or ex-husband or ex-partner pushed, hit, slapped, kicked, choked, or physically hurt them in any other way 12 months before or during the most recent pregnancy; the variable was further recoded and a dichotomous variable was created “no” or “at least one event”.

Finally, to account for state tobacco control policies, like regulatory intervention, we included the “Tobacco 21 Grade” as a measure of contextual and structural tobacco control factors. This grade assesses how effectively each U.S. state implements and enforces the minimum legal age of 21 for purchasing tobacco products. Developed by the Preventing Tobacco Addiction Foundation, the Tobacco 21 grade evaluates state laws based on several criteria, including enforcement strength, retailer licensing, penalties for violations, and coverage of all tobacco and nicotine products. States receive letter grades ranging from A (strongest) to F (weakest), reflecting their adherence to these best practices. States that do not have a statewide Tobacco 21 policy are classified as having “No policy” [27].

### 2.4. Statistical Analysis

The primary aim of this analysis was to examine trends in perinatal smoking (e.g., smoking persistence) from 2009 to 2021 (Sample 1), stratified by area of residence (rural vs. urban). We calculated weighted prevalence estimates and corresponding 95% confidence intervals (CIs) by year and area of residence. To assess temporal trends while accounting for regional differences, we used logistic regression models with a Wald test for significance. An interaction term between year of delivery and area of residence (UrbanRural_YYInteraction) was included to evaluate whether smoking trends differed between urban and rural areas.

To explore structural and intermediary factors that may explain the association between perinatal smoking and area of residence, we performed a bivariate analysis between perinatal smoking and explanatory variables (e.g., age, race/ethnicity, marital status, education, household income, WIC participation, pregnancy experience, and exposure to perinatal stressors), using Sample 2 (PRAMS Phase 8, 2016–2021). Variable selection for multivariable modeling was guided by these bivariate associations and prior literature on smoking during pregnancy. We assessed multicollinearity using Variance Inflation Factor (VIF) and Tolerance Statistics. Model fit was evaluated using Pseudo R-Square values.

To investigate disparities in smoking cessation by area of residence, we performed similar analyses on a subsample of smokers (Sample 3). Missing data were less than 10%, overall; thus, listwise deletion was applied. For variables with more than 2% missing data (e.g., household income, 9.1%), a “missing” category was created and included in the models. All analyses applied PRAMS weights to account for the complex sampling design and nonresponse, using IBM SPSS Complex Samples Version 29.0 (IBM Corp., Armonk, NY, USA) [28]. Statistical significance was set at *p* < 0.05. The manuscript followed the STROBE guidelines for reporting observational studies.

## 3. Results

### 3.1. Participant Characteristics

Table S2 (Supplementary Material) presents perinatal smoking patterns and population characteristics stratified by urban and rural residency for sample 1. Table 1 depicts sociodemographic, health outcomes, pregnancy-related characteristics, and psychosocial factors of mothers residing in urban (*N* = 182,923) and rural areas (*N* = 49,485) for Sample 2. Compared to urban mothers, rural mothers were generally younger (6.3% vs. 3.8%, *p* < 0.001), more likely to be non-Hispanic White (73.9% vs. 53.6%, *p* < 0.001), not married (42.7% vs. 37.4%, *p* < 0.001), and had fewer years of education (12 yrs.: 31.6% vs. 23.4%, *p* < 0.001). A higher proportion of rural mothers participated in the WIC program (43.4% vs. 31.9%, *p* < 0.001) and reported experiencing more perinatal depression (18.3% vs. 13.0%, *p* < 0.001) and perinatal stressful life events compared to urban mothers (5.0% vs. 3.3%, *p* <0.001). Finally, regarding state control policies (Tobacco 21 Grade), rural areas were more likely to have weaker policies, with higher proportions of F grades (31.3%) and no policy at all (16.7%) than urban areas (19.7% and 9.3%, respectively).

### 3.2. Trends in the Prevalence of Perinatal Smoking Behavior by Rural–Urban Residence

**Figure 2:**
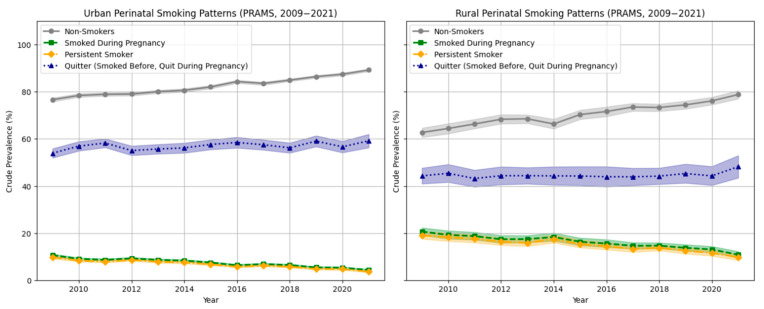
Trends in smoking cessation and perinatal smoking (during pregnancy and smoking persistence) by rural and urban status, PRAMS, 2009 to 2021. Significant linear trend: *p* < 0.001. Non-smoker: did not smoke before, during, or after pregnancy. Smoked during pregnancy: smoked during the last 3 months of pregnancy. Persistent smoking: Smoked before, during, and after pregnancy. Quitter: Smoked before, quit during pregnancy. Unweighted N = 493,455.

Figure 2 shows trends in smoking patterns (crude prevalence) among pregnant women, stratified by rural–urban residency, from 2009 to 2021. Smoking patterns included: *non-smokers* (did not smoke at any point: before, during, and after pregnancy), *smoked during pregnancy* (last 3 months of pregnancy), *persistent smoker* (smoked before, during, and after pregnancy), and *quitter* (smoked before but not during pregnancy). During the study period, the prevalence of never smoking across the 3 periods of pregnancy increased in both urban (from 76.6% to 89.2%) and rural areas (from 62.7% to 78.8%) between 2009 and 2021. At the same time, the rates of smoking during pregnancy and persistent smoking significantly declined in both settings (*p* < 0.001). However, despite this decline, persistent smoking remained more prevalent in rural areas (9.8%, 95% CI: 8.5–11.1) in 2021 compared to urban areas (3.7%, 95% CI: 3.3–4.0).

**Figure 3:**
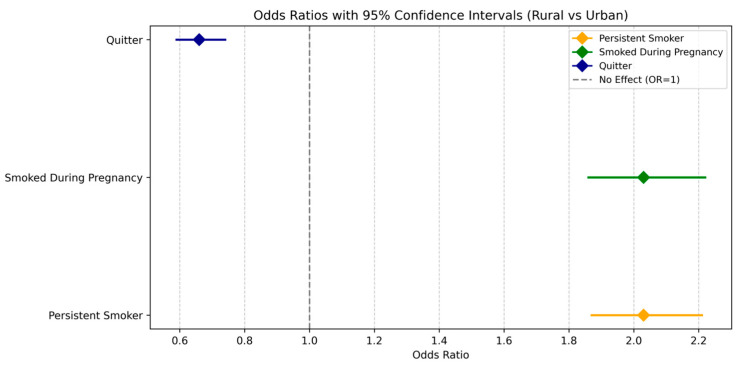
Regression analysis: Adjusted Odds ratio of cigarette perinatal smoking (during pregnancy, quitter, and smoking persistence) and persistent smoking by rural and urban status, and 95% confidence bars PRAMS, from 2009 to 2021. Model adjusted for birth (birth year/year of delivery), region, interaction term (birth year × rural–urban area), Rural Vs. Urban. Smoking persistence: Defined as any cigarette smoking during all three periods (reference category = No). Smoking cessation: Smoked before, quit during pregnancy (reference = No). Any cigarette smoking during the last 3 months of pregnancy (reference = No). Unweighted N = 493,455.

After adjusting for regional differences and including the interaction between rural–urban status and birth year, the interaction term was significant for both persistent smoking (Wald F = 3.549, *p* < 0.001) and smoking during pregnancy (Wald F = 2.95, *p* < 0.001). This indicates that trends over time in both outcomes differed significantly between rural and urban pregnant women. Specifically, pregnant women in rural areas experienced a slower and less consistent decline in persistent smoking compared to their urban counterparts, with an adjusted odds ratio of 0.89 (95% CI: 0.87–0.92) per year. In contrast, for smoking cessation, the interaction between rural–urban status and the birth year was not statistically significant (Wald F = 0.984, *p* = 0.475), indicating no statistical difference in cessation trends between rural and urban areas over the study period. The overall trend in cessation was stable (Wald F = 2.929, *p* = 0.087), and the OR associated with birth year was 1.03 (95% CI: 1.00–1.07). Additionally, as visualized in Figure 3, pregnant women in rural areas had significantly higher odds of persistent smoking (OR = 2.03, 95% CI: 1.86–2.22) and significantly lower odds of smoking cessation (OR = 0.66, 95% CI: 0.59–0.74) compared to their urban counterparts, while adjusting for regional differences. These findings highlight the rural–urban disparities in perinatal smoking behaviors from 2009 to 2021.

### 3.3. Explaining the Association Between Perinatal Smoking Behaviors and Rural–Urban Residence

Table 2 shows the results of weighted logistic regression analyses, presenting the unadjusted and adjusted odds ratios (aOR) for smoking patterns by rural–urban status. The association between perinatal smoking and rural–urban residency was statistically significant. Rural mothers were more than twice as likely to engage in persistent smoking compared to urban mothers (OR = 2.61, 95% CI: 2.46, 2.77). After adjusting for structural and intermediary health factors, such as maternal income, education, WIC program participation, and exposure to perinatal stressors, the multivariable logistic regression model indicated that rural mothers remained 45% more likely to smoke persistently throughout pregnancy compared to urban mothers (aOR = 1.45, 95% CI: 1.35, 1.56). This highlights the influence of structural and intermediary factors on maternal smoking. For instance, a social gradient was evident, with lower income and education levels being associated with higher odds of persistent smoking.

Regarding smoking cessation, rural women were 26% less likely to quit smoking during pregnancy compared to their urban counterparts (OR = 0.74, 95% CI: 0.67–0.80). Furthermore, states without a “Tobacco 21” policy were 20% less likely to see smoking cessation compared to states with an “A” grade policy.

Sensitivity analyses, including both complete and missing case scenarios, produced similar estimates, confirming the robustness of the findings.

## 4. Discussion

This study analyzed trends in perinatal smoking among pregnant women in rural and urban areas of the U.S. from 2009 to 2021. It also investigated whether structural and intermediate health factors contributed to observed geographic disparities. Our findings show a persistent decline in perinatal smoking in both rural and urban areas over time. Nevertheless, rural mothers showed a slower decline and remained significantly more likely to be persistent smokers and less likely to quit during pregnancy than their urban counterparts. Additionally, in 2021, only 89.1% of rural mothers reported abstaining from smoking during pregnancy, compared to 95.6% of urban mothers. These findings indicate that rural areas are lagging behind in meeting the Healthy People 2030 goal of 95.7% abstinence [29]. Structural and intermediary health factors, including socioeconomic status, access to social programs (e.g., WIC), psychosocial stressors, regional differences, and tobacco control policies, only partially explained these disparities.

Our findings are consistent with previous studies reporting poorer health outcomes and higher smoking rates in rural settings, including among pregnant women [10,11,25,30,31,32]. Although some studies found an inverse association [9] between smoking and rural–urban status, they focused on different population groups. To the best of our knowledge, research specifically examining perinatal smoking by rural–urban residents remains limited, especially in non-U.S. contexts. By providing updated trend data and accounting for multiple determinants, our study contributes new insights into the persistence of rural–urban disparities in perinatal smoking, including the post-pandemic period.

The overall decline in perinatal smoking likely reflects the influence of successful tobacco control policies, public health campaigns, and the evolving social norms regarding smoking, particularly its harms during pregnancy. However, our findings suggest that rural mothers still face structural barriers to cessation and higher nicotine dependence, and that tobacco control efforts may vary across rural settings. Studies have suggested that rural areas often face weaker tobacco control environments, including lower policy grades, higher retail tobacco density, and limited enforcement of smoke-free laws [8,10,17]. In addition, cultural norms surrounding secondhand smoke (SHS) exposure [17,33] may contribute to persistent disparities. Previous research has shown that rural residents were less supportive of SHS-related policies, being 22% less likely to restrict smoking at home and 13% less likely to support car smoking bans [33]. This lower policy support may contribute to ongoing SHS exposure during pregnancy, which is associated with increased risks of stillbirth, low birth weight, congenital malformations [34], and postpartum smoking relapse [35]. Hence, culturally sensitive approaches that engage household members, especially partners, are essential to reducing SHS exposure and encouraging cessation.

Despite no change in smoking cessation rates over time in both rural and urban areas, rural mothers were less likely to quit smoking; a similar finding was reported by Parker et al. [12]. The challenges in the healthcare infrastructure and limited access to technology in rural areas may contribute to this issue. For instance, underfunded public health programs and a shortage of healthcare infrastructure and providers in rural areas have been previously reported [18,26,36,37,38]. It has been shown that while rural areas may allocate a higher percentage of their tax base to public health, their per capita public health funding remains lower. Additionally, only 10% of healthcare professionals serve rural communities, limiting access to cessation counseling and related services during prenatal care [26]. Besides these challenges, innovation in cessation services is also needed. Evidence-based interventions, such as opt-out referrals, carbon monoxide (CO) monitoring [39], peer-led support, and financial incentives [40], have been shown to provide positive results and thus could be expanded in rural areas. Telemedicine and programs like WIC could also serve as platforms to deliver these strategies in a cost-effective and accessible manner [41].

### 4.1. Limitations

This study has several strengths, including a large sample representative of the PRAMS sites within the study period, as well as its emphasis on various stages of perinatal smoking and cessation. It also includes contextual, structural, and intermediary health determinants such as the Tobacco 21 policy, income, education, psychosocial factors, and access to assistance programs. However, the findings from this study have limitations. The cross-sectional design prevents inference of causality. The use of self-reported tobacco data, instead of objective measures (e.g., biomarkers such as cotinine [42]), may lead to the underestimation of smoking prevalence due to recall bias or social desirability bias, biasing the magnitude of the association. While PRAMS captures e-cigarette use in Phase 8, this study focused solely on self-reported cigarette smoking for consistency across survey years. The binary classification of rural and urban areas may oversimplify the variability within highly rural regions, a limitation noted in previous studies [43,44]. Additionally, PRAMS does not collect data on migration status, which further limits the generalizability of the results. While the overall response rate of the survey remained stable in 2020, it declined in 2021 (PRAMS), likely due to broader societal and operational challenges following the pandemic. The design also prevents longitudinal analyses, limiting our ability to track changes within individuals. Lastly, unmeasured confounders, such as SHS exposure throughout pregnancy and access to cessation resources, may have impacted the results.

### 4.2. Implications and Future Directions

Efforts to reduce perinatal smoking should prioritize culturally tailored, evidence-based cessation programs for rural populations, while also addressing the unique barriers faced in these areas. Patient and Public Involvement (PPI) can enhance the development of interventions that are responsive to and aligned with community needs [45]. However, more research is needed to understand the cultural and contextual drivers of perinatal smoking in rural areas [46]. Longitudinal and multilevel studies may be particularly beneficial for this purpose. Additionally, future tobacco cessation programs could greatly benefit from being co-designed with local communities. These programs should include messaging tailored for rural areas, culturally competent counseling, and involvement with trusted rural stakeholders, such as rural health clinics and community-based organizations.

## 5. Conclusions

This study presents updated information on perinatal smoking and cessation while stratifying by rural–urban status in the U.S. While perinatal smoking rates have decreased in both groups, significant disparities between rural and urban areas remain. A comprehensive approach that combines effective tobacco control strategies with interventions tailored to the specific needs of pregnant women in rural areas could help address these gaps. However, more research is needed to clarify the underlying factors contributing to these differences.

## Figures and Tables

**Table 1:** Descriptive characteristics of the aggregated 2016–2021 sample of pregnant women by rural versus urban area of residency, PRAMS (unweighted N = 232,408).

Variable	Category	Unweighted N	Overall % ^a^ (95% CI)	Urban % ^a^ (95% CI)	Rural % ^a^ (95% CI)
Smoking status					
Before pregnancy	Yes	39,441	15.5 (15.2–15.7)	13.7 (13.5–14.0)	24.9 (24.2–25.6)
During pregnancy	Yes	19,280	7.1 (6.9–7.3)	5.9 (5.7–6.0)	13.8 (13.2–14.4)
After pregnancy	Yes	26,358	10.0 (9.8–10.2)	8.6 (8.4–8.8)	17.6 (17.0–18.3)
Persistent	Yes	17,367	6.4 (6.2–6.5)	5.2 (5.1–5.4)	12.6 (12.1–13.1)
Cessation	Yes	20,326	54.6 (53.7–55.4)	57.8 (56.8–58.7)	44.9 (43.2–46.5)
Maternal age (yrs.)	<20	10,330	4.2 (4.1–4.3)	3.8 (3.7–4.0)	6.3 (6.0–6.7)
	20–24	41,169	18.0 (17.8–18.3)	16.6 (16.4–16.9)	25.7 (25.0–26.4)
	25–29	66,363	28.7 (28.4–29.0)	28.0 (27.7–28.4)	32.6 (31.9–33.4)
	30–34	69,641	30.0 (29.8–30.3)	31.3 (30.9–31.6)	23.3 (22.7–24.0)
	≥35	44,896	19.0 (18.7–19.2)	20.3 (20.0–20.5)	12.1 (11.6–12.5)
	Missing	9	0.0 (0.0–0.0)	0.0 (0.0–0.0)	0.0 (0.0–0.0)
Race/Ethnicity	White	104,681	56.7 (56.4–57.0)	53.6 (53.3–53.9)	73.9 (73.2–74.5)
	Black	41,396	15.0 (14.8–15.2)	16.2 (16.0–16.5)	8.1 (7.6–8.5)
	Hispanic	40,954	18.0 (17.8–18.2)	19.4 (19.1–19.7)	10.3 (9.9–10.8)
	Other ^b^	39,179	9.4 (9.3–9.6)	10.0 (9.8–10.2)	6.5 (6.1–6.7)
	Missing	6198	0.9 (0.8–0.9)	0.8 (0.7–0.8)	1.3 (1.3–1.4)
Marital status	Married	139,066	61.7 (61.4–62.0)	62.5 (62.2–62.8)	57.2 (56.4–58.0)
	other	93,156	38.3 (38.0–38.6)	37.4 (37.1–37.8)	42.7 (41.9–43.5)
	Missing	186	0.1 (0.0–0.1)	0.0 (0.0–0.1)	0.1 (0.1–0.2)
Education (yrs)	<12	27,944	11.6 (11.4–11.8)	11.1 (10.9–11.3)	14.1 (13.6–14.7)
	12	55,825	24.6 (24.3–24.9)	23.4 (23.0–23.7)	31.6 (30.9–32.4)
	>12	146,638	63.0 (62.7–63.3)	64.7 (64.3–65.0)	53.8 (53.0–54.6)
	Missing	2001	0.8 (0.8–0.9)	0.9 (0.8–0.9)	0.5 (0.4–0.6)
Household income (US$)	≤$24,000	70,727	28.4 (28.1–28.7)	27.0 (26.7–27.4)	35.7 (35.0–36.5)
	$24,001–60,000	60,649	25.6 (25.3–25.9)	24.6 (24.3–24.9)	31.0 (30.3–31.7)
	>$60,001	79,183	37.0 (36.6–37.2)	39.0 (38.7–39.4)	25.4 (24.7–26.0)
	Missing	21,849	9.1 (8.9–9.3)	9.3 (9.1–9.5)	7.9 (7.5–8.4)
Received WIC	Yes	83,337	33.6 (33.3–33.9)	31.9 (31.5–32.2)	43.4 (42.6–44.1)
	No	145,727	65.0 (64.7–65.3)	66.7 (66.4–67.1)	55.7 (54.9–56.5)
	Missing	3344	1.3 (1.3–1.3)	1.4 (1.3–1.5)	0.9 (0.8–1.1)
Parity	0	90,641	38.8 (38.5–39.2)	39.4 (39.0–39.7)	35.9 (35.2–36.7)
	1	72,802	32.8 (32.5–33.1)	33.0 (32.7–33.3)	31.5 (30.8–32.2)
	2	38,356	16.5 (16.2–16.7)	16.1 (15.9–16.4)	18.1 (17.5–18.7)
	≥3	30,173	11.8 (11.5–12.0)	11.3 (11.1–11.5)	14.3 (13.8–14.9)
	Missing	436	0.2 (0.2–0.2)	0.2 (0.2–0.2)	0.1 (0.1–0.2)
Pregnancy intention	Planned	99,561	44.9 (44.4–44.4)	44.4 (44.1–44.8)	42.1 (41.4–42.9)
	Mistimed	76,229	33.0 (32.7–33.4)	33.2 (32.9–33.6)	32.1 (31.3–32.8)
	Unsure	52,934	21.3 (21.0–21.6)	20.8 (20.5–21.0)	24.4 (23.7–25.1)
	Missing	3684	1.6 (1.5–1.7)	1.6 (1.5–1.7)	1.4 (1.3–1.6)
Prenatal depression	Yes	160,357	85.2 (85.0–85.4)	86.0 (85.7–86.2)	80.8 (80.2–81.5)
	No	22,997	13.8 (13.6–14.0)	13.0 (12.8–13.2)	18.3 (17.7–19.0)
	Missing	2437	1.0 (0.9–1.1)	1.0 (1.0–1.1)	0.8 (0.7–1.0)
Perinatal stressors ^c^	No	222,777	96.4 (96.3–96.5)	96.7 (96.5–96.8)	95.0 (94.6–95.3)
	Yes	9631	3.6 (3.5–3.7)	3.3 (3.2–3.5)	5.0 (4.7–5.4)
Region	Midwest	59,428	25.1 (25.0–25.2)	23.3 (23.2–23.4)	35.1 (34.5–35.8)
	Northeast	58,871	26.2 (26.2–26.3)	28.9 (28.7–29.0)	11.8 (11.3–12.4)
	South	55,747	32.8 (32.7–32.9)	31.6 (31.4–31.7)	36.3 (35.6–37.0)
	West	58,362	16.3 (16.3–16.4)	16.3 (16.2–16.3)	16.8 (16.4–17.1)
Tobacco 21 Grade	A	39,168	18.9 (18.8–19.0)	20.8 (20.7–20.9)	8.5 (8.1–8.9)
	B	26,218	10.1 (10.1–10.2)	10.2 (10.1–10.3)	9.5 (9.0–9.9)
	C	75,271	39.0 (38.9–39.1)	39.9 (39.8–40.1)	34.0 (33.3–34.7)
	F	65,510	21.5 (21.4–21.5)	19.7 (19.6–19.8)	31.3 (30.7–31.9)
	No policy	26,241	10.5 (10.4–10.5)	9.3 (9.3–9.4)	16.7 (16.3–17.2)

Abbreviations: CI, confidence interval; WIC, Special Supplemental Nutrition Program for Women, Infants, and Children. ^a^ Values are weighted percent. ^b^ Other races include those who self-identified as American Indian/Alaskan Native, Asian/Pacific Islander, Chinese, Japanese, Filipino, Hawaiian, and Other Asian. ^c^ Perinatal stressors (Intimate partner violence) include physical or verbal abuse by husband/partner or ex-husband/partner, or the husband/partner experienced 12 months before or during pregnancy. Unweighted N = 182,923 (Urban), 49,485 (Rural).

**Table 2:** Unadjusted and adjusted logistic regression coefficients for a model of smoking persistence across pregnancy and smoking cessation, Pregnancy Risk Assessment Monitoring System (PRAMS), 2016, 2021.

Variable	Category	Smoking Persistence (N = 221,236)		Smoking Cessation (N = 37,547)	
		Unadjusted OR (95% CI)	aOR (95% CI)	Unadjusted OR (95% CI)	aOR (95% CI)
Residency	Rural	2.61 (2.46–2.77)	1.45 (1.35–1.56)	0.59 (0.55–0.64)	0.74 (0.67–0.80)
	Urban	ref	ref	ref	ref
Maternal age	<20	1.56 (1.35–1.81)	0.30 (0.25–0.36)	1.34 (1.13–1.60)	0.32 (0.27–0.39)
	20–24	1.95 (1.78–2.13)	0.59 (0.53–0.67)	1.08 (0.97–1.21)	0.64 (0.58–0.72)
	25–29	1.70 (1.57–1.85)	0.91 (0.83–1.01)	0.92 (0.83–1.02)	0.99 (0.90–1.08)
	30–34	1.15 (1.05–1.25)	1.02 (0.92–1.13)	1.02 (0.91–1.14)	1.08 (0.98–1.19)
	≥35	ref	ref	ref	ref
Race/ Ethnicity	White	1.94 (1.77–2.11)	2.45 (2.21–2.71)	0.84 (0.75–0.93)	2.38 (2.16–2.63)
	Black	1.26 (1.13–1.40)	0.58 (0.52–0.66)	0.96 (0.84–1.10)	0.57 (0.50–0.64)
	Hispanic	0.41 (0.35–0.47)	0.22 (0.18–0.25)	1.89 (1.61–2.20)	0.24 (0.21–0.28)
	Missing	1.35 (1.08–1.67)	1.25 (0.97–1.61)	0.96 (0.71–1.30)	1.11 (0.88–1.42)
	Other	ref	ref	ref	ref
Marital status	Married	ref	ref	ref	ref
	Other	4.71 (4.44–4.99)	2.73 (2.53–2.95)	2.74 (2.55–2.95)	2.74 (2.55–2.95)
Education (yrs)	<12	4.23 (3.93–4.54)	2.97 (2.69–3.27)	0.35 (0.31–0.38)	2.92 (2.66–3.20)
	12	3.42 (3.22–3.64)	1.81 (1.68–1.96)	0.55 (0.51–0.60)	1.84 (1.71–1.97)
	>12	ref	ref	ref	ref
Household income (US$)	≤$24,000	12.87 (11.63–14.24)	5.38 (4.70–6.16)	0.21 (0.19–0.24)	0.32 (0.27–0.39)
	$24,001–$60,000	4.90 (4.39–5.46)	2.94 (2.60–3.33)	0.39 (0.35–0.44)	0.49 (0.43–0.57)
	Missing	4.35 (3.79–4.98)	2.90 (2.46–3.41)	0.30 (0.26–0.35)	0.40 (0.33–0.49)
	>$60,001	ref	ref	ref	ref
Received WIC	Yes	3.08 (2.92–3.26)	1.30 (1.21–1.39)	0.55 (0.51–0.58)	0.82 (0.76–0.89)
	No	ref	ref	ref	ref
	Zero	ref	ref	ref	
Parity	1	1.39 (1.30–1.49)	1.43 (1.32–1.55)	0.61 (0.56–0.66)	0.65 (0.59–0.71)
	2	2.00 (1.85–2.16)	1.66 (1.51–1.83)	0.45 (0.41–0.50)	0.56 (0.50–0.62)
	3+	2.86 (2.65–3.10)	1.91 (1.72–2.12)	0.28 (0.26–0.32)	0.40 (0.35–0.45)
Pregnancy intention	Planned	ref	ref	ref	ref
	Mistimed	1.49 (1.39–1.60)	1.21 (1.11–1.30)	0.88 (0.81–0.96)	0.90 (0.82–0.99)
	Unsure	3.58 (3.36–3.83)	1.75 (1.62–1.89)	0.55 (0.51–0.60)	0.75 (0.68–0.83)
Prenatal Depression	Yes	5.13 (4.70–5.60)	2.08 (1.94–2.23)	0.56 (0.52–0.60)	0.71 (0.66–0.78)
	No	ref	ref	ref	ref
Perinatal stressors	Yes	2.02 (1.81–2.26)	2.02 (1.81–2.26)	0.52 (0.46–0.58)	0.69 (0.61–0.79)
	No	ref	ref	ref	ref
Region	Midwest	1.58 (1.47–1.69)	1.25 (1.15–1.36)	0.76 (0.69–0.82)	0.83 (0.75–0.92)
	Northeast	0.98 (0.90–1.07)	1.19 (1.07–1.33)	0.97 (0.88–1.07)	0.86 (0.76–0.98)
	South	1.23 (1.15–1.33)	1.07 (0.98–1.17)	0.89 (0.81–0.97)	0.99 (0.89–1.11)
	West	ref	ref	ref	ref
Tobacco 21 Grade	A	ref	ref	ref	ref
	B	1.65 (1.48–1.85)	1.25 (1.11–1.39)	0.82 (0.71–0.94)	0.93 (0.79–1.10)
	C	1.51 (1.38–1.65)	1.24 (1.09–1.41)	0.77 (0.69–0.86)	0.87 (0.76–0.99)
	F	1.79 (1.63–1.97)	1.41 (1.23–1.62)	0.74 (0.66–0.83)	0.92 (0.79–1.06)
	No policy	2.21 (1.99–2.44)	1.25 (1.11–1.39)	0.61 (0.54–0.69)	0.79 (0.67–0.92)

aOR = adjusted odds ratio.

## Data Availability

The data presented in this study are available under request to the CDC PRAMS at https://www.cdc.gov/prams/php/data-research/index.html, accessed on 1 June 2023. Data received in June 2023.

## Supplementary Materials

The following supporting information can be downloaded at https://www.mdpi.com/article/10.3390/ijerph22060895/s1, Table S1: Measurements of perinatal smoking; Table S2: Sample characteristics, PRAMS, 2009–2021.
