# Supplementary material for: Rural–Urban Disparities in Perinatal Smoking in the United States: Trends and Determinants
Source: Int J Environ Res Public Health. 2025 Jun 4;22(6):895. doi: 10.3390/ijerph22060895 (PMC12193116; doi:10.3390/ijerph22060895)
Supplement: Supplementary file 1 [file ijerph-22-00895-s001.zip › ijerph-3502477-supplementary.pdf]

International Journal of
Environmental Research and Public Health

MDPI

Supplemental Material

Table S1. Measurements of perinatal smoking.

**Non-smoker:** Did not smoke before, during, or after pregnancy.
**Persistent Smokers**: Smoked before, during, and after pregnancy.
**Quitter/cessation**: Smoked 3 months before, quit during pregnancy.
**Smoked before pregnancy** = smoked 3 months before pregnancy
**Smoked during pregnancy** = smoked during the last 3 months of pregnancy
**Smoked after pregnancy** = smoked during the postpartum period (assessed at the time the questionnaire was completed (2-6 months after delivery)

Table S2. Sample characteristics, PRAMS, 2009-2021.

**Characteristic**	**Category**	**Urban % (95% CI)**	**Rural % (95% CI)**	**Total % (95% CI)**	**Unweighted N**
Smoking Pattern	Non-smoker	82.5% (82.3-82.7)	70.6% (70.1-71.1)	80.7% (80.5-80.9)	388532
	Persistent Smoker	6.7% (6.6-6.9)	14.9% (14.5-15.3)	8.0% (7.8-8.1)	47218
Maternal Age	<20	5.4% (5.3-5.6)	8.3% (8.0-8.6)	5.9% (5.8-6.0)	30783
	20-24	18.5% (18.3-18.7)	27.2% (26.8-27.6)	19.8% (19.7-20.0)	98852
	25-29	28.4% (28.2-28.7)	32.2% (31.8-32.6)	29.0% (28.8-29.2)	140805
	30-34	29.4% (29.2-29.6)	21.5% (21.1-21.9)	28.2% (28.0-28.4)	137055
	35+	18.2% (18.0-18.4)	10.8% (10.5-11.1)	17.1% (16.9-17.3)	85941
Marital Status	Married	62.1% (61.9-62.3)	57.4% (57.0-57.8)	61.4% (61.2-61.6)	294359
	Other	37.9% (37.7-38.1)	42.6% (42.2-43.0)	38.6% (38.4-38.8)	198273
Race/Ethnicity	White, non-Hispanic	55.1% (54.9-55.3)	75.9% (75.5-76.3)	58.2% (58.0-58.4)	233870
	Black, non-Hispanic	15.5% (15.4-15.7)	6.6% (6.4-6.8)	14.2% (14.0-14.4)	82726
	Hispanic	19.7% (19.5-19.9)	11.1% (10.9-11.3)	18.4% (18.2-18.6)	83417
	Other	9.6% (9.5-9.8)	6.4% (6.2-6.6)	9.1% (8.9-9.3)	78550
Region	Midwest	25.9% (25.8-26.0)	36.8% (36.4-37.2)	27.5% (27.3-27.7)	123767
	Northeast	29.3% (29.2-29.4)	12.8% (12.6-13.0)	26.9% (26.7-27.1)	123370
	South	29.1% (29.0-29.2)	34.0% (33.7-34.3)	29.8% (29.6-30.0)	119475
	West	15.8% (15.7-15.9)	16.5% (16.3-16.7)	15.9% (15.7-16.1)	126843
Year of Birth	2009	8.2% (8.1-8.3)	8.9% (8.8-9.0)	8.3% (8.2-8.4)	39736
	2010	8.4% (8.3-8.5)	7.4% (7.3-7.5)	8.2% (8.1-8.3)	39160

2011	6.6% (6.5-6.7)	6.0% (5.9-6.1)	6.5% (6.4-6.6)	37279
2012	6.9% (6.8-7.0)	6.7% (6.6-6.8)	6.8% (6.7-6.9)	32655
2013	6.5% (6.4-6.6)	6.8% (6.7-6.9)	6.6% (6.5-6.7)	38430
2014	6.1% (6.0-6.2)	6.0% (5.9-6.1)	6.1% (6.0-6.2)	33133
2015	9.3% (9.2-9.4)	8.6% (8.5-8.7)	9.2% (9.1-9.3)	40654
2016	8.1% (8.0-8.2)	6.8% (6.7-6.9)	7.9% (7.8-8.0)	35320
2017	7.5% (7.4-7.6)	7.8% (7.7-7.9)	7.6% (7.5-7.7)	36860
2018	8.3% (8.2-8.4)	9.9% (9.8-10.0)	8.5% (8.4-8.6)	42892
2019	9.0% (8.9-9.1)	9.4% (9.3-9.5)	9.0% (8.9-9.1)	43441
2020	8.2% (8.1-8.3)	8.1% (8.0-8.2)	8.2% (8.1-8.3)	40142
2021	6.9% (6.8-7.0)	7.7% (7.6-7.8)	7.0% (6.9-7.1)	33753

PRAMS sites included in the analysis = 48 (AK, AL, AR, AZ, CO, CT, DC, DE, FL, GA, HI, IA, IL, IN, KS, KY, LA, MA, MD, ME, MI, MN, MO, MS, MT, NC, ND, NE, NH, NJ, NM, NY, OH, OK, OR, PA, RI, SD, TN, TX, UT, VA, VT, WA, WI, WV, WY, YC)AL, AR, AZ, CO, CT, DC, DE, FL, GA, HI, IA, IL, IN, KS, KY, LA, MA, MD, ME, MI, MN, MO, MS, MT, NC, ND, NE, NH, NJ, NM, NY, OK, OR, PA, RI, SD, TN, TX, UT, VA, VT, WA, WI, WV, WY, YC)
